# Marshall H. Webb, D. D. S.

**Published:** 1883-03

**Authors:** H. C. Longnecker


### ARTICLE V.

### *Marshall H. Webb, D. D. S.*

DIED, at Lancaster, Pa., January 1, 1882, of cancer of the colon, Dr. Marshall H. Webb, in the thirty-ninth year of his age.

---

Dr. Webb's death was not unexpected, the fact of his serious illness having been known for months. It was, and is, however, a subject of gratitude that, although confined to his bed for the last year, he did not at any time through all his long and tedious illness suffer severe physical pain.

Marshall Hickman Webb was born at Marlborough, Chester Co., Pa., October 28, 1844. After the completion of his elementary education, he became, in 1861, a student of Dr Frank Hickman's, who was at that time engaged in the practice of dentistry at Coatesville, Pa.,—Prof. Taylor, principal of Chester Valley Academy, assisting him in his general education. He made rapid progress, and showed distinguished ability in both his professional and academical studies. Having completed his term of pupilage with Dr. Hickman, he attended lectures at the Philadelphia Dental College, and graduated from that institution in 1867. Immediately after, he commenced his professional career at Lancaster, Pa.

Dr. Webb's consummate skill in operative dentistry and his thorough knowledge of dental pathology, soon gained for him a very extensive practice, and brought his name prominently before the dental profession. He was a member of the Harris Dental Association of Lancaster County, the Pennsylvania State Dental Society, the American Dental Association, the New York Odontological Society and the Odontological Society of Pennsylvania. He was a lecturer on operative destistry and dental histology in the Dental Department of the University of Pennsylvania. He was honorary member of many dental societies, and a delegate to the Medical Congress that assembled in London, 1881.

Dr. Webb was not an ordinary man. By his professional brethren he was regarded as one of the brightest, most energetic, and withal most self-sacrificing workers in the dental profession. His ambition was to place operative dentistry upon a higher plane, and attain this end no sacrifice seemed to him too great. His work was at all times marked with a sincere purpose and an honest conviction.

To the student he was ever ready to lend a helping hand and no one of his years ever had a greater number of private pupils or more ardent and zealous followers. All of his pupils will recall many acts attesting his interest in their advancement, and will say with one accord that they owe Dr. Webb a debt of gratitude for his unselfish devotion.

As a clinical instructor he stood without a peer, gentle but firm, always ready, even eager to demonstrate practically the ideas which he advocated in published essays from time to time; as a student he was earnest and thorough; as a practitioner concientious, capable, and faithful.

He infused life and enthusiasm among his fellows, and was a hearty worker for the success and advancement of the societies of which he was a member. His efforts in anything he undertook were marked with persistent energy, and though not blessed with a strong physical organism, he was nevertheless, capable of an immese amount of work.

No man toiled harder than Dr. Webb, and probably no one has done so much to elevate the standard of operative dentistry. His operations were faultless in point of execution, and there was an elegance about their finish that was truly fascinating. The skill which he attained gave him a prominence in his profession surpassed by none, and though dying so young he was fairly entitled to rank as one of the most distinguished of American dentists.

Dr. Webb had a love of fun and a fondness for caricature which he indulged without bitterness or cynicism. His arrows were sharp, were aimed with an honest motive, and always hit the mark. His target was the pretender, and no hand has done more than his in so few years of labor to expose shams in the profession and lift up genuine merit to its rightful place. He has written nothing which will not help to make better dentists and better men. It may not be amiss to add that the last work of Dr. Webb is now passing through the press. The manuscript was prepared during his months of illness, which did not in the least affect his clear intellect. The dental student or practitioner who has not read Dr. Webb's contributions to dental literature should by all means do so. His beautiful methods will commend themselves to every thoughtful practitioner, and he will learn to value thorough operations and to despise slovenly performances more than ever before.

Of Dr. Webb's home life; of his hospitality; of his relations as husband and father, it need only be said he xemplified everywhere and always the traits of a Christain gentleman. Dear, kind-hearted, good-natured Webb! Who is there to take his place?

Dr. Webb left a widow and three children to mourn his loss, a clientage which will not forget his faithfulness, a social circle to which he was endeared, and a profession which will honor itself in honoring his memory.

H. C. L.

We have laid aside a biographical notice of Dr. Webb in order to admit one prepared by his former pupil and friend, Dr. Longnecker; but the occasion demands something more than a passing tribute to Dr. Webb's disinterested and valuable services to the profession. He gave time and effort without stint to teach all who chose to learn whatever improved methods he had devised, and it is safe to say that all over the country better dentistry is being done to-day in many offices as the result of Dr. Webb's clinics and teaching. A sad side of the case is that, with the feeling that life was before him—years of labor,—he gave more of his time and strength for the general good than he could afford to; more doubtless than he would have done had he foreseen the early temination of his career. He has left a widow and three children unprovided for. His record is finished and is before the profession. Already, without solicitation, a score of operators have expressed their sense of obligation and their sympathy by generous subscriptions to a testimonial fund. One of these—an operator of note—remarked in subscribing, "If that pays for what I learned of Dr. Webb it was the cheapest tuition I ever received; it is not simply a pleasure to contribute, but under the circumstances a duty. Many beside myself ought to recoginize the substantial advantages which will accure to them as the result of Dr. Webb's instructions, and which will continue as long as they remain in practice."

The editor of the *Dental Cosmos* has been solicited to act as treasurer, and will gladly receive and acknowledge any subscriptions which may be sent as a token of appreciation of the unselfish work of Dr. Webb and of sympathy with his bereaved family.—*Dental Cosmos.*

